# Biodegradation of gentamicin by bacterial consortia AMQD4 in synthetic medium and raw gentamicin sewage

**DOI:** 10.1038/s41598-017-11529-x

**Published:** 2017-09-08

**Authors:** Yuanwang Liu, Huiqing Chang, Zhaojun Li, Yao Feng, Dengmiao Cheng, Jianming Xue

**Affiliations:** 1 Institute of Agricultural Resources and Regional Planning, Chinese Academy of Agricultural Sciences, Key Laboratory of Plant Nutrition and Fertilizer, Ministry of Agriculture, Beijing, 100081 China; 2 Henan University of Science and Technology, Luoyang, 471003 China; 3 Scion, Christchurch, 29-237 New Zealand

## Abstract

Gentamicin, a broad spectrum antibiotic of the aminoglycoside class, is widely used for disease prevention of human beings as well as animals. Nowadays the environmental issue caused by the disposal of wastes containing gentamicin attracts increasing attention. In this study, a gentamicin degrading bacterial consortia named AMQD4, including *Providencia vermicola*, *Brevundimonas diminuta*, *Alcaligenes* sp. and *Acinetobacter*, was isolated from biosolids produced during gentamicin production for the removal of gentamicin in the environment. The component and structure of gentamicin have a great influence on its degradation and gentamicin C1a and gentamicin C2a were more prone to being degraded. AMQD4 could maintain relatively high gentamicin removal efficiency under a wide range of pH, especially in an alkaline condition. In addition, AMQD4 could remove 56.8% and 47.7% of gentamicin in unsterilized and sterilized sewage in a lab-scale experiment, respectively. And among the isolates in AMQD4, *Brevundimonas diminuta* BZC3 performed the highest gentamicin degradation about 50%. It was speculated that *aac3iia* was the gentamicin degradation gene and the main degradation product was 3′-acetylgentamicin. Our results suggest that AMQD4 and *Brevundimonas diminuta* BZC3 could be important candidates to the list of superior microbes for bioremediation of antibiotic pollution.

## Introduction

Gentamicin is a broad spectrum aminoglycoside antibiotic and China is a country with the largest gentamicin output in the world^1^. By the very nature of the case, a large amount of wastes containing gentamicin produced during gentamicin production and course of practical application in hospital and livestock farm were poured into the environment, which can induce development of gentamicin resistance genes^2–4^. The problem of drug resistance could reduce the function of antibiotics. Furthermore the large amount of gentamicin production solid waste and livestock excrements containing gentamicin have also been limited to be used as organic fertilizer. So far, few effective methods were exploited to deal with the gentamicin residues. Thus, it is critically urgent to develop economically feasible solutions for effectively removing or reducing the gentamicin residues in wastes and the environment.

The heat stable characteristic of gentamicin and its resistance to both acidic and alkaline conditions make it a big challenge to remove gentamicin from the environment using common chemical degradation or physical methods. Bioremediation, in contrast, is an attractive and successful cleaning technique for polluted environment^5^. And it is commonly considered that chemicals may be easily degraded by microflora via complementary transformation reactions^6^. Actually, microbial biodegradation and bioremediation technology has been increasingly applied to removal of antibiotics from the environment^7^. Selvi *et al*. isolated a kind of fungi named *Trametesversicolor* which could remove ciprofloxacin and norfloxacin more than 90%^8^. Islas-Espinoza *et al*. found that a consortium including *Bacillus licheniformis*, *Pseudomonas putida*, *Alcaligenes* sp. and *Aquamicrobium defluvium* could enhance the degradation of sulfonamides in soil^9^. However, the degradation of antibiotics depends on the various involved functional microflora^10^. So it is indispensable to isolate specific microorganism or consortia for different antibiotics. To our knowledge, thus far, few of previous studies were conducted for the research about bioremediation of gentamicin or used for the treatment of raw gentamicin sewage.

In recent years, along with the development of sequencing technique, comparative genomics was more used in the study of functional genes, especially the virulence genes and resistance genes^11, 12^. To provide information on gentamicin degradation genes and to evaluate its destruction mechanism, comparative genomics sequencing technique was used in this study. In this way more useful information including other kinds of antibiotics degradation genes and resistance genes could be further tapped.

In this case we supposed to develop an efficient microbial technology which will be applied for gentamicin removal from the environment. In consideration of the stability of consortia, this study was planning to domesticate and isolate effective gentamicin-degrading microflora. After the optimization of fermentation conditions, the removal ability of the microflora in raw gentamicin sewage was determined for the large-scale usage. In addition, the degradation ability of the isolated bacteria from the microflora was detected and the degradation genes and gentamicin destruction mechanism were identified and evaluated using comparative genomics sequencing technique.

## Results

### isolating of gentamicin-degrading bacterial consortia

**Figure 1:**
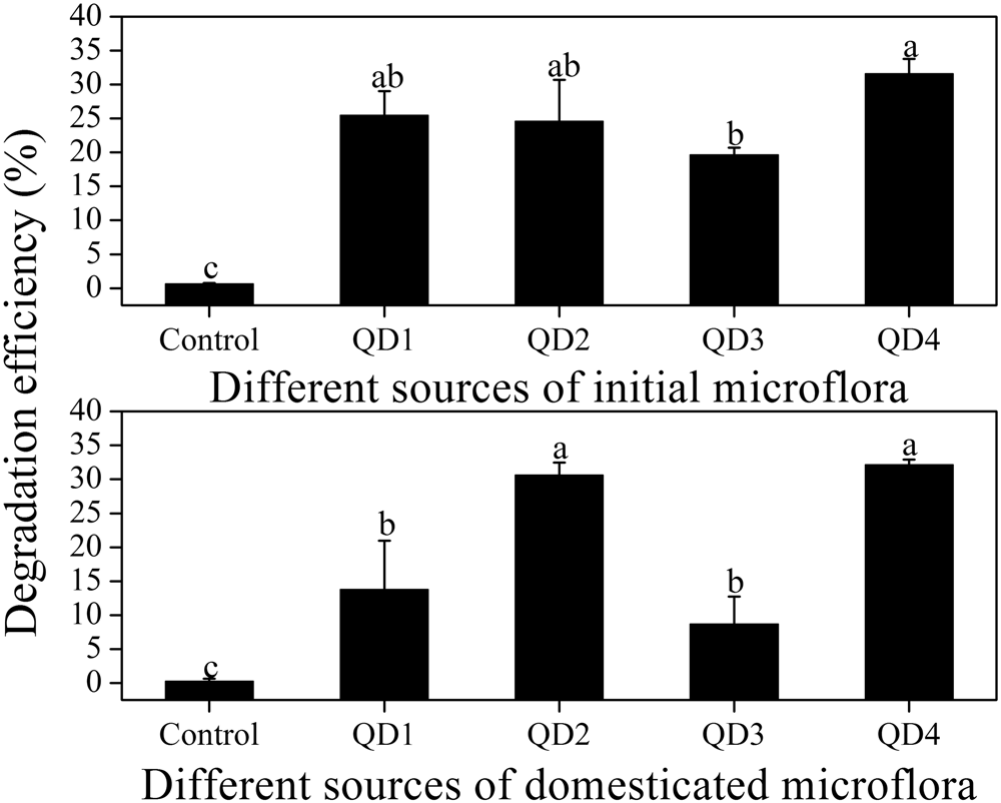
Degradation efficiencies of gentamicin by initial and domesticated microflora isolated from different sources of wastes (QD1, fermentation sludge; QD2, sludge maintained in anaerobic jar; QD3, sludge maintained in aerobic jar; bio-solids QD4). Condition: 1/5 BEP with 100 mg L^−1^ gentamicin incubated at 30 °C and 150 r/min orbital shaking. The mean values and SD (*error bars*) from triplicate trials are presented. *Data bars* having the same letter are not significantly different from each other at the 95% confidence level in the Duncan’s test (P < 0.05).

As shown in Fig. 1, both degradation efficiencies of gentamicin in the 1/5 of beef extract peptone medium (BEP) inoculated with initial and domesticated microflora were significantly higher than those in controls (P < 0.05). The initial bacteria consortia before domestication from fermentation waste water (QD1), anaerobic jar sludge (QD2), aerobic tank sludge (QD3) and bio-solids sludge (QD4) could degrade gentamicin by 25.5%, 24.5%, 19.6% and 31.6% of initial gentamicin content, respectively. The degradation efficiency of acclimatized ones from QD1 and QD3 decreased to 13.7% and 8.6%, respectively. Hence these two types of microflora (QD1 and QD3) were not chosen in the following experiments due to low gentamicin degradation ability. In contrast, the degradation efficiency of mixed bacteria from QD2 and QD4 increased to 30.5% and 32.2%, respectively. Because of the higher degradation efficiency, acclimatized microflora in QD4 (AMQD4) was considered for further degradation studies. In addition, no significant changes were found for optical density of medium at 600 nm (OD600) and gentamicin contents in MSM (data not shown) over the domestication period.

### Optimization of gentamicin degradation by AMQD4

**Figure 2:**
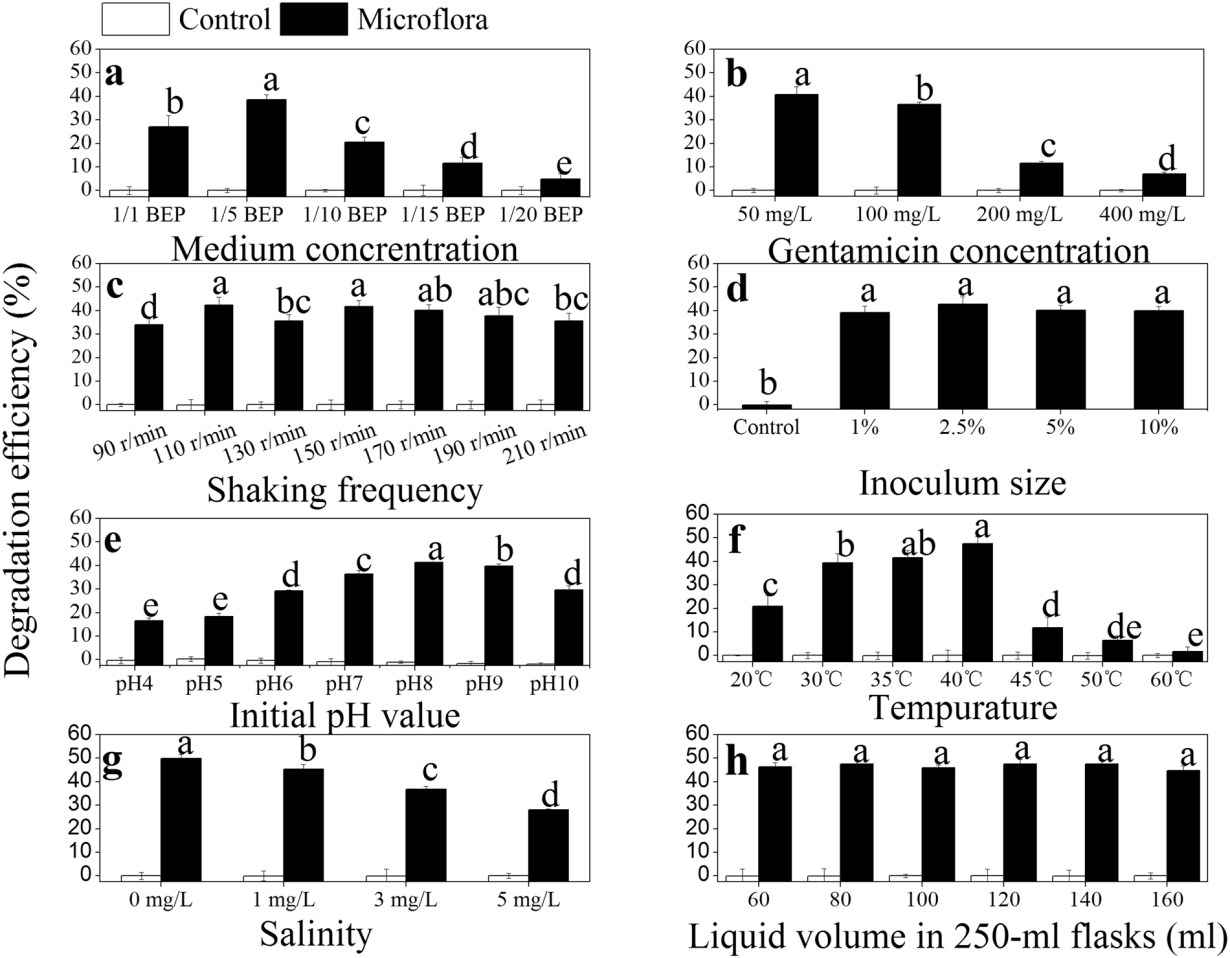
The effect of different culture conditions on the gentamicin degradtion by AMQD4 at the end of fermentation ((**a)**, medium concentration; (**b**), gentamicin concentration; (**c**), shaking frequency; (**d**), inoculum size; (**e**), initial pH value; (**f**), temperature; (**g**), salinity; (**h**), liquid volume). The mean values and SD (*error bars*) from triplicate trials are presented. *Data bars* having the same letter are not significantly different from each other at the 95% confidence level in the Duncan’s test (P < 0.05).

The medium concentrations had significant (P < 0.05) effects on the gentamicin degradation by AMQD4 (Fig. 2a). The maximum gentamicin degradation efficiency with a value of about 38.6% was obtained in 1/5 of BEP medium. The degradation efficiency of gentamicin by AMQD4 decreased when the medium was diluted from 1/5 to 1/20 of BEP (Fig. 2a). In addition, pH values and bacterial growth (OD600) at the end of fermentation decreased significantly (P < 0.05) from 9.1 to 7.9 (Fig. S1a) and from 4.0 to 0.12 (Fig. S2a), respectively, when the medium was diluted from 1/1 to 1/20 of BEP.

At the optimized medium concentration of 1/5 of BEP, the degradation efficiency of gentamicin by AMQD4 decreased with concentration of gentamicin increasing in the medium (Fig. 2b). For example, the gentamicin degradation efficiencies were significantly (P < 0.05) increased from 7.0% to 40.8% when the gentamicin concentrations in the medium were reduced from 400 mg L^−1^ to 50 mg L^−1^. It was found that AMQD4 could grow well and reach the largest biomass (OD600 = 0.64) in the medium containing 100 mg L^−1^ of gentamicin and could keep active growth (OD600 = 0.45) even in medium with high dose of gentamicin (400 mg L^−1^) (Fig. S2b). It is interesting to note that the final pH values of the medium at the end of fermentation were significantly increased from 8.4 to 8.9 along with the decrease of gentamicin concentration from 400 mg L^−1^ to 50 mg L^−1^ (P < 0.05) (Fig. S1b).

Shaking frequency also significantly (P < 0.05) affected the gentamicin degradation by AMQD4 **(**Fig. 2c **)**. The microflora had greater gentamicin degradation efficiencies at 110 and 150 r/min with the values of 42.4%, and 41.8%, respectively. However, it is observed that the gentamicin degradation efficiency decreased to 35.6% when the shaking frequency was increased from 150 to 210 r/min. What is interesting is that the pH in spent medium almost did not rise as the shaking frequency increased from 150 to 210 r/min (Fig. S1c). As shown in Fig. S2c, the biomass of AMQD4 reached the maximum with the OD600 value of 0.66 at 130 r/min.

The gentamicin degradation efficiency didn’t vary significantly with the inoculum size of AMQD4 **(**Fig. 2d **)**. It appeared that the gentamicin degradation efficiency was slightly higher in the culture medium inoculated with 2.5% (2.05 × 10^9^ CFU) of AMQD4 than those inoculated with 1%, 5% and 10% of AMQD4. It is also observed that 10% of inoculation quantity gave the both the highest OD600 and pH with the value of 0.62, and 8.84, respectively. However, the lowest values of both OD600 and pH were obtained when 2.5% of AMQD4 was added (Figs S1 d and S2d).

The gentamicin degradation efficiencies significantly (P < 0.05) increased with increasing pH from 4 to 8, but decreased afterwards (Fig. 2e **)**. The maximum degradation efficiency with a value of 41.4% was obtained at initial pH 8. Interestingly, the final pH of the spent medium increased from 8.4 to 8.9 as initial pH increased from 4 to 10 **(**Fig. S1e). However, the most suitable initial pH for AMQD4 growth appeared at pH5 with an OD600 value of 0.66 (Fig. S2e).

The gentamicin degradation efficiency increased significantly (P < 0.05) with increasing temperature from 20 to 40 °C, with a maximum value of 47.4% at 40 °C, which was 2.4-fold greater than that at 20 °C (Fig. 2f). However, the gentamicin degradation efficiency decreased dramatically (P < 0.05) from 40 to 60 °C, with values of 11.7% at 45 °C and even only 1.7% at 60 °C. The pattern of final pH change was similar to that of the gentamicin degradation efficiency (Fig. S1f **)**, with the greatest pH value of 9.4 at 40 °C and the lowest one of 7.8 at 60 °C. OD600 significantly (P < 0.05) increased from 0.38 at 20 °C to the maximum value of 0.56 at 40 °C and then sharply decreased to 0.02 at 45 °C, with no differences afterwards (Fig. S2f).

The degradation efficiency of gentamicin decreased significantly (P < 0.05) with increasing concentrations of salinity, with the maximum value of 49.8% without NaCl in the medium **(**Fig. 2g). The final pH values were greater at the higher addition (3 and 5 mg L^−1^) than lower addition (0 and 1 mg L^−1^) of NaCl (Fig. S1g **)**. In contrast, higher OD600 values were achieved at lower addition of NaCl (0 and 1 mg L^−1^) (Fig. S2g).

The various liquid volumes from 60 to 160 ml (in 250-ml flasks) had no significant effect on the gentamicin degradation efficiency (Fig. 2h **)**. The final pH of the spent medium gradually decreased (Fig. S1h **)** while OD600 values gradually increased **(**Fig. S2h **)** with increasing liquid volume from 60 to 160 ml.

**Figure 3:**
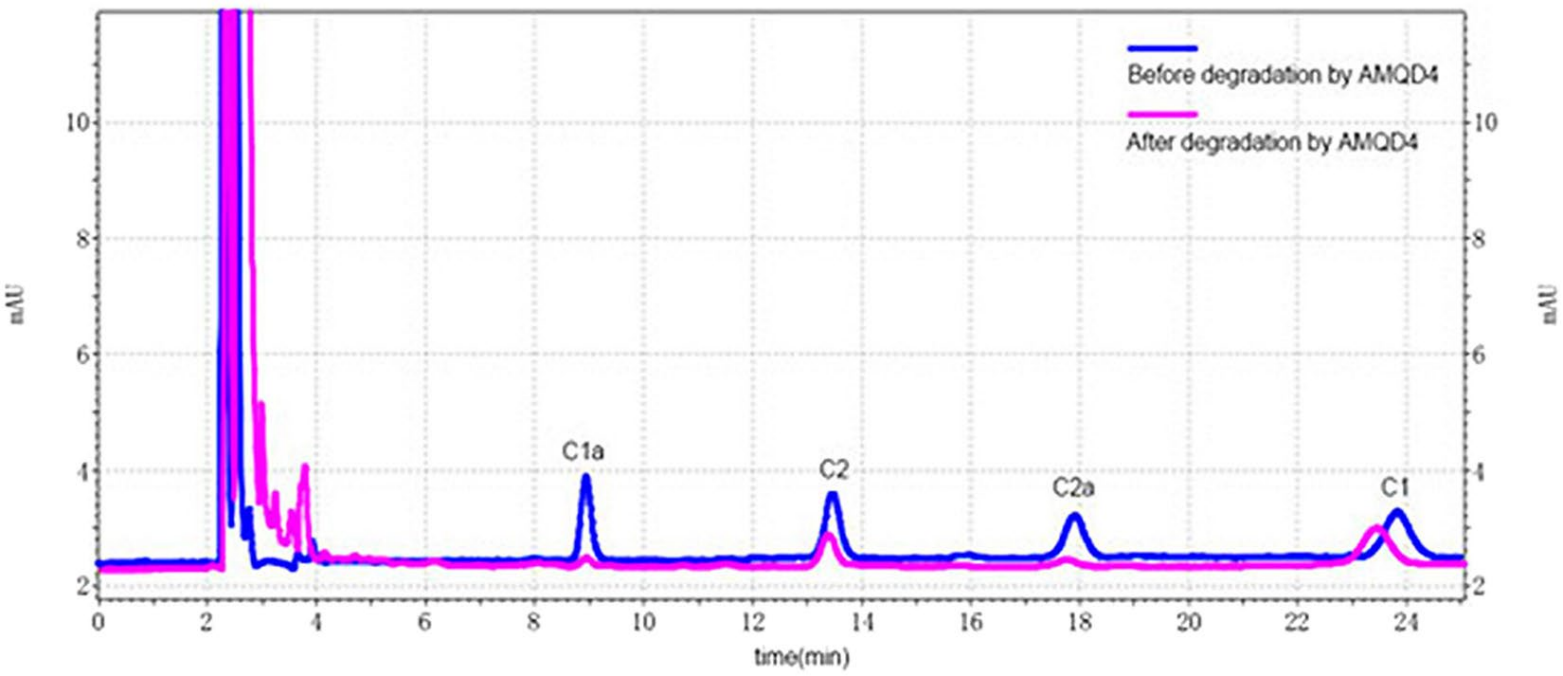
The chromatogram of 50 mg/L of gentamicin before and after degradation by AMQD4.

In addition, the chromatogram of 50 mg/L of gentamicin before and after degradation by AMQD4 was shown in Fig. 3. Obviously, the components named gentamicin C1a (C1a) and gentamicin C2a (C2a) were more prone to being degraded than those named gentamicin C1 (C1) and gentamicin C2 (C2).

### Application of AMQD4 in the degradation of gentamicin in raw gentamicin sewage

**Figure 4:**
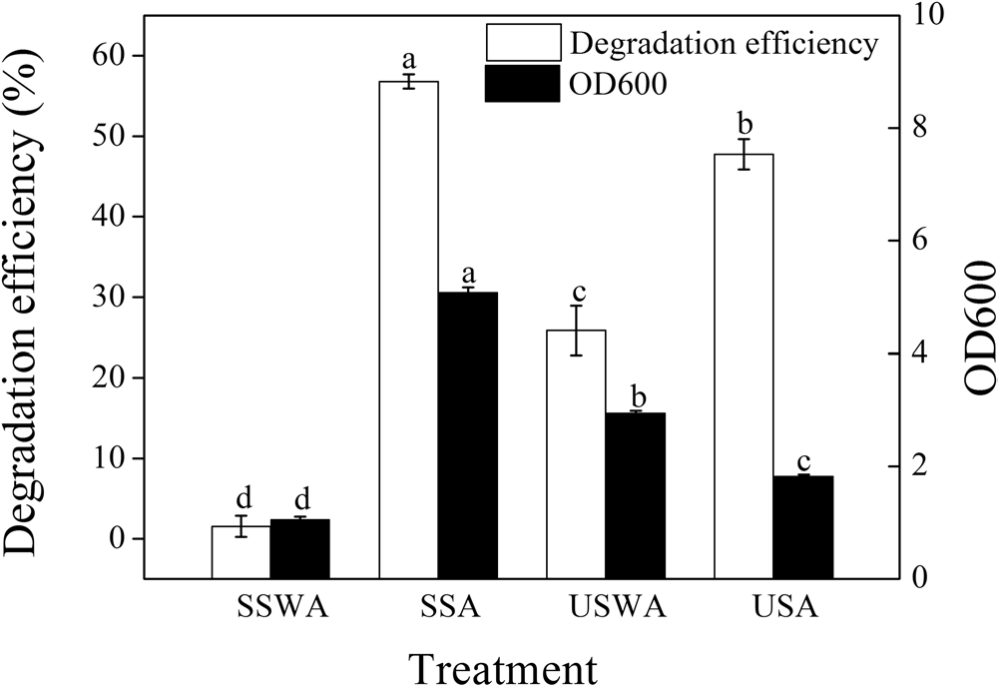
The effects of AMQD4 on the degradation and microbial biomass in gentamicin sewage (SSWA, sterilized sewage without AMQD4; SSA, sterilized sewage +2.5% AMQD4; USWA, unsterilized sewage without AMQD4; (4) USA, unsterilized sewage +2.5% AMQD4). The mean values and SD (*error bars*) from triplicate trials are presented. *Data bars* having the same letter are not significantly different from each other at the 95% confidence level in the Duncan’s test (P < 0.05).

To further confirm the role of AMQD4 in the degradation of gentamicin, a lab-scale sewage treatment experiment was conducted. According to the results shown in Fig. 4, the addition of AMQD4 had significant effects on the gentamicin degradation and microbial biomass. When the sterilized sewage was inoculated with 2.5% AMQD4, the maximum value of degradation efficiency reached to 56.8%, 37.8 times higher than that of SSWA. And the maximum value of biomass reached to 5.1, 4.8 times higher than that of SSWA. The biomass of USWA, with a value of 2.9, was 1.6 times higher than that of USA. However, gentamicin degradation efficiency of the later was 47.7%, which was 1.8 times higher than that of the former.

### Identification of the isolated bacteria and their gentamicin degradation efficiencies

**Figure 5:**
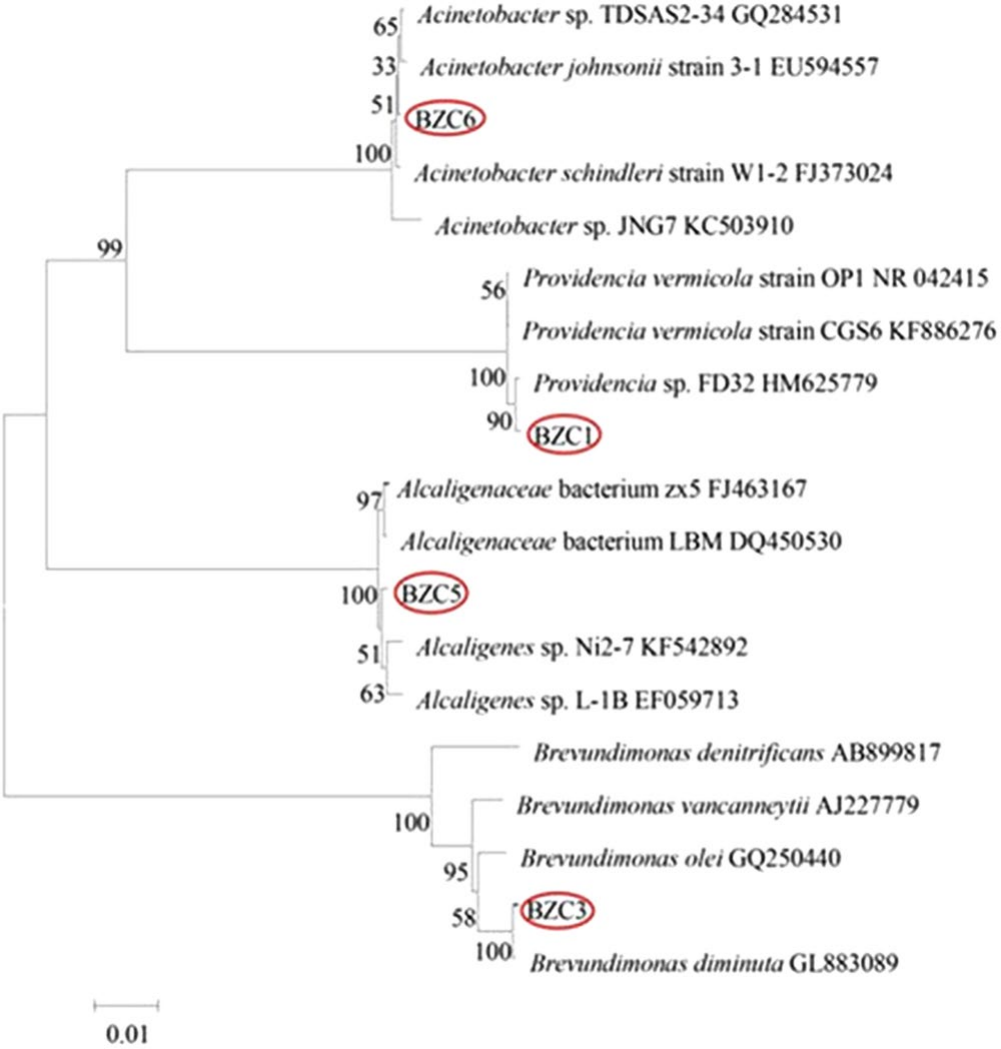
Phylogenic tree of the four isolated bacterial strains (BZC1, BZC3, BZC5, BZC6) in the consortia of AMQD4 and other relatives within genus based on 16 rRNA gene sequence.

Originally, seven bacterial isolates named as BZC1-7 were found to compose the gentamicin degrading microflora AMQD4. By comparing the 16S rDNA sequences with public reference ones in NCBI database, it was found that BZC1, BZC2, BZC4, and BZC7 have the same 16S rDNA sequences and that the 16S rDNA sequences of BZC1, BZC3, BZC5, and BZC6 are different from each other. A phylogenetic tree for them and their close relatives (≥99%) was conducted in MEGA6 using the neighbor-joining method and was shown in Fig. 5. According to the sequence similarity and evolutionary analyses, they were identified as *Providencia vermicola*, *Brevundimonas diminuta*, *Alcaligenes* sp. and *Acinetobacter* respectively.

**Figure 6:**
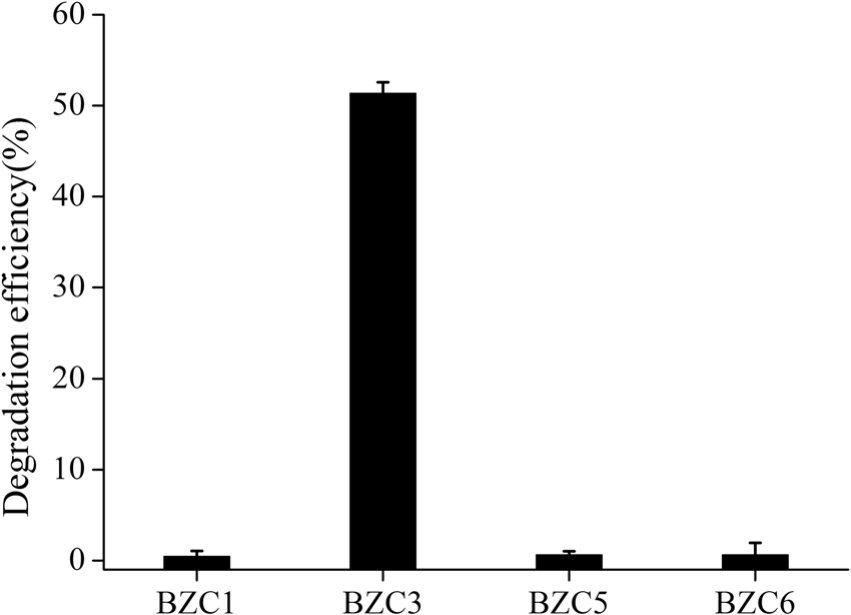
The gentamicin degradation efficiencies of the four bacteria isolated from AMQD4. The mean values and SD (*error bars*) from triplicate trials are presented. *Data bars* having the same letter are not significantly different from each other at the 95% confidence level in the Duncan’s test (P < 0.05).

Gentamicin degradation efficiencies of the four isolates are shown in Fig. 6. Obviously, BZC3 performed the highest degradation efficiency about 50%, while the other three bacteria showed little gentamicin degradation.

### Identification of degradation genes

Some general genome properties of *Brevundimonas diminuta* BZC3 and *Brevundimonas diminuta* ACCC10507 are shown in Table S1. The fine genome of BZC3 was constructed with one scaffold containing 6 contigs. The draft genome of ACCC10507 was constructed with 57 scaffolds containing 57 contigs. The genome size and coding sequences (CDS) of BZC3 is slightly smaller but similar to that of ACCC10507. The G + C contents of the two strains were high to 67.3%, similar to the what was reported by Ghosh *et al*.^13^.

**Figure 7:**
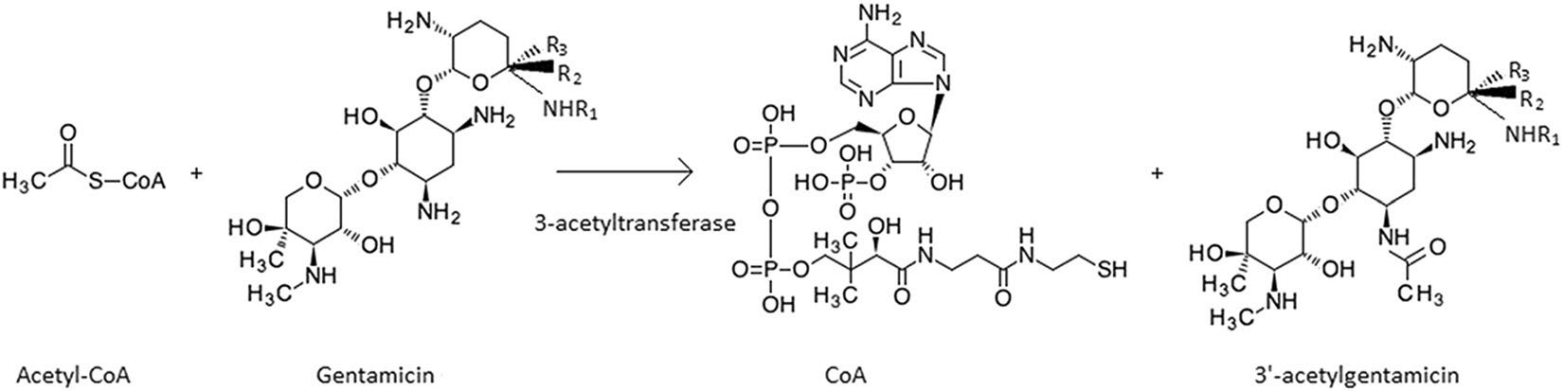
The equation of gentamicin degradation by *Brevundimonas diminuta* BZC3.

The antibiotic resistance genes annotated by Antibiotic Resistance Genes Database (ARDB) is shown in Table 1. By comparing the resistance genes existed in BZC3 and ACCC10507, it was found that the particular ones in BZC3 were *aac3iia*, *mexe* and *oprm*. *Mexe* and *oprm* are multidrug resistance efflux pump. And *aac3iia* is a gene coding aminoglycoside 3-acetyltransferase, which modify aminoglycosides by acetylation and is responsible for the resistance of gentamicin, netilmicin, tobramycin, sisomicin, dibekacin. So it was speculated that *aac3iia* was the gentamicin degradation gene and the main reaction product was 3′-acetylgentamicin (Fig. 7).

**Table 1:** The antibiotic resistance genes annotated by ARDB.

Resistance genes	Antibiotic resistance	Description
*dfra26*	trimethoprim	Group A drug-insensitive dihydrofolate reductase, which can not be inhibited by trimethoprim.
*emre*	aminoglycoside	Multidrug resistance efflux pump.
aac3iia*	gentamicin, netilmicin, tobramycin, sisomicin, dibekacin	Aminoglycoside N-acetyltransferase, which modifies aminoglycosides by acetylation.
*mexf*	chloramphenicol, fluoroquinolone	Resistance-nodulation-cell division transporter system. Multidrug resistance efflux pump.
*ceoa*	chloramphenicol	Resistance-nodulation-cell division transporter system. Multidrug resistance efflux pump.
mexe*	chloramphenicol, fluoroquinolone	Resistance-nodulation-cell division transporter system. Multidrug resistance efflux pump.
oprm*	aminoglycoside, tigecycline, fluoroquinolone, beta_lactam, tetracycline	Resistance-nodulation-cell division transporter system. Multidrug resistance efflux pump.
*baca*	bacitracin	Undecaprenyl pyrophosphate phosphatase, which consists in the sequestration of Undecaprenyl pyrophosphate.
*ykkd*	na_antimicrobials	Small Multidrug Resistance (SMR) protein family. Multidrug resistance efflux pump, which consists of two proteins.
*catb3*	chloramphenicol	Group B chloramphenicol acetyltransferase, which can inactivate chloramphenicol. Also referred to as xenobiotic acetyltransferase.
*bl2e_y56*	cephalosporin	Class A beta-lactamase. This enzyme breaks the beta-lactam antibiotic ring open and deactivates the molecule’s antibacterial properites.
*teta*	tetracycline	Major facilitator superfamily transporter, tetracycline efflux pump.

*The genes only exist in *Brevundimonas diminuta* BZC3.

## Discussion

This is the first study, to our knowledge, to remove gentamicin, a broad spectrum antibiotic of the aminoglycoside class and also a kind of hazardous material to non-targeted organisms, through domesticating and isolating an appropriate bacterial consortium. Usually, the domestication is carried out in a solid or liquid medium with constant content of nutrient and toxicant^14^, which could not provide a gradual process for the microorganisms to adjust to more harsh circumstance. This is not profitable for the promotion of microbial ability to degrade gentamicin. The present domestication was conducted following a progressive method, *i*.*e*. the microflora was continuously cultured in the same type of medium, in which, however, the gentamicin concentration increased and nutrient concentration decreased at every medium renewal. This progressive domestication, which could gradually promote the adaptive capacity of target microorganisms to worse environment, was proved to be effective and enabled us to isolate the novel bacterial consortia of AMQD4, including *Providencia vermicola*, *Brevundimonas diminuta*, *Alcaligenes* sp. and *Acinetobacter*, for enhanced degradation of gentamicin in submerged fermentation. An important characteristic of AMQD4 is that each bacterial member of the consortia was able to eliminate toxic compounds in the environment^9, 15, 16^. In addition, in consideration of that AMQD4 was isolated from bio-solids sludge, which is a kind of waste including a great deal of gentamicin residues, AMQD4 could adapt to the environments containing gentamicin and maintain effective gentamicin degradation.

It has been reported that microbial population generally has intimate connection with the bioremediation of a contaminant^17^. However, the patterns of degradation efficiencies (Fig. 3) and cell density (i.e. OD600) (Fig. S2) changes over the culture period in this study indicated that the removal of gentamicin by AMQD4 was not completely growth-associated. This is in agreement with a previous study on the degradation of sulfamethoxazole by mixed bacteria^18^. The uncoupling of degradation and biomass in this study might be related to the different peak biomass growth for each bacterial member of AMQD4 or the uneven gentamicin degradation abilities of them, which masked the relationship between the degradation efficiency and biomass growth. On the other hand, this uncoupling relationship implies that the degradation efficiency of gentamicin could be more dependent on the total microbial activity (than total biomass growth), which was determined by the activities of each bacterial member of AMQD4 and their relative contribution.

So far antibiotic could be degraded by photolysis, cathode, metal salt, hydrolysis and microorganism^19–22^. In this study, no significant changes of gentamicin contents were found in the control treatment, excluding the possible of non-microbial degradation. Microorganisms usually degrade pollutants by co-metabolism or using them as growth-substrates^23^. The fact that AMQD4 did not grow in MSM and gentamicin contents did not change significantly proved that the microflora could not use gentamicin as sole carbon source. While enhanced degradation of gentamicin by AMQD4 in 1/5 diluted medium indicated that AMQD4 removed gentamicin through cometabolism. Cometabolism is a better way to deal with contaminants because even exposed in the environment (i.e. with carbon source) the pollutants could be rapidly degraded by microorganisms^23^.

Besides the culture medium, the amount of pollutants in the submerged fermentation is another vital factor that affects the pollutant removal efficiency through impacting microbial metabolic activity and growth^24^. The greater gentamicin removal efficiency (by AMQD4) was achieved at the lower initial concentrations of gentamicin (50–100 mg L^−1^) in this study, implying that microbial activity could be inhibited by higher concentrations of gentamicin, similar to the report on the biodegradation of oxytetracycline by *Pleurotusostreatus* mycelium^5^.

Agitation has been known to be an important factor for toxicant degradation by microorganism because of its effect on substrate, heat and dissolved oxygen transfers^25^. Generally, higher shaking frequency can enhance the bioremediation of pollutants in aerobic fermentation because of more oxygen availability. The greater gentamicin degradation efficiency at a relatively lower stirring level (110 r/min) could be related to the facultative anaerobic characteristic of the related species such as *Alcaligenes* sp. and *Providencia vermicola*. In fact, the relatively lower stirring could benefit for saving the energy used for the gentamicin bioremediation in the future.

Inoculum size is an important parameter that affects biodegradation efficiency of targeted contaminant as well as microbial growth. Selvi *et al*. found that the biodegradation efficiency of cefdinir depended on the inoculum dosage of functional bacterial strain and the more microorganism was added, the more antibiotic was reduced^8^. Unlike the standpoint above, our study showed that the difference in inoculum size had no significant impacts on the gentamicin removal, indicating that small inoculum dosage was also effective for bioremediation of gentamicin.

The initial pH value of the medium is a pivotal factor affecting enzymes production, which usually plays a key role in degrading organic contaminant^8, 14^. In this study, the AMQD4 could survive and maintain relative high degradation efficiency in a wide range of initial pH from 4.0 to 10.0, which is an important implication for future management of wastes produced from the gentamicin production industry for pollution control. The final pH range of 8.4 to 8.9 and highest degradation with the initial pH of 8.0 may suggest that an initial pH range of 8.0 to 9.0 is optimum for gentamicin degradation. The similar result was obtained by Jyoti *et al*. when *Pseudomonas diminuta* was used for biodegradation of methyl parathion^26^. The better degradation effects of pollutants in alkaline medium may result from that the contributing enzymes secreted by the relative microorganism keep a higher activity in alkaline environment^27^.

Due to its effects on microbial growth, enzymes production, cell structure and cell membrane, etc, temperature is usually considered as one of the most critical element among the eco-physiological factors influencing the bioremediation of toxicants. Generally, the optimum range of temperature for biodegradation of pollutants is from 30 to 35 °C^8, 28, 29^. Similarly, the greatest degradation efficiency of gentamicin by the AMQD4 was obtained at 35–40 °C in this study. The sharp decrease of the gentamicin degradation at higher temperature (i.e. >40 °C) was closely associated with the concurrent decrease of microbial density, indicating the inhibition of higher temperature on the gentamicin degradation was through its effect on microbial growth. The variation of gentamicin removal, final pH and OD600 followed the same tendency, which may result from the mesophilic nature of AMQD4, especially the effect of high temperature on the growth of BZC5 (*Alcaligenes* sp.).

In addition, salinity is another factor influencing the degradation of pollutants significantly. Similar to what has been found by Jyoti *et al*.^26^, in this study, the decreased degradation efficiency of gentamicin with increasing salinity was at least partially attributed to the adverse impacts of salt ions on microbial density and/or cellular metabolism. And the fact that higher OD600 values were achieved at lower addition of NaCl may result from the favorable effect of salt on the growth of *Alcaligenes* sp. and adverse effect on other three bacteria which may play more important role on gentamicin removal than *Alcaligenes* sp.

Another factor affecting the biodegradation of gentamicin by AMQD4 is the components and structure of gentamicin. C1a and C2a were almost removed completely, but the degradation efficiencies of C1 and C2 were much lower (Fig. 3). The perssad R_3_ of C1a and C2a are both –H and the one of C1 and C2 are both –CH_3_, suggesting that R_3_ could affect the degradation of gentamicin.

AMQD4 showed a positive effect on the degradation of gentamicin in sterilized or unsterilized sewage. Plentiful substrates are used during the process of gentamicin production by *Micromonospora* species, which contributes to abundant nutrition in the gentamicin sewage^30^. So the AMQD4 could grow well in sewage, but better in unsterilized sewage than in sterilized sewage, indicating that AMQD4 and the native microflora inhibit the growth of one another. Even so, the addition of AMQD4 in unsterilized sewage promoted the degradation of gentamicin. This may foreshadow the potential of AMQD4 for future use in large-scale biodegradation of gentamicin in raw gentamicin sewage.

It is common thought that chemicals are prone to be mineralized by mixed consortia of bacteria via complementary transformation reactions^6, 18^. However, by detecting the gentamicin degradation efficiencies of the isolates obtained from AMQD4, it was found that *Brevundimonas diminuta* BZC3 performed higher gentamicin degradation efficiency than AMQD4. Another same species’s failing to degrade gentamicin proved that the gentamicin degradation ability of *Brevundimonas diminuta* BZC3 may be the results of environmental filtering and gene mutation. Aminoglycoside 3-acetyltransferase coded by *aac3iia* could modify amino on gentamicin, which confers resistance to gentamicin^31^. This could inactivate gentamicin to target microorganisms and reduce its toxicity. In addition, according to the annotation by ARDB, some other kinds of antibiotics degradation genes, such as *bl2e_y56* coding beta-lactamase, have been found. This enzyme breaks the beta-lactam antibiotic ring open and deactivates the molecule’s antibacterial properties. So more useful functions could be mined from genomic sequencing of *Brevundimonas diminuta* BZC3 and *Brevundimonas diminuta* ACCC10507.

## Materials and Methods

### Chemicals

Gentamicin (purity, 99.99%), composed of C1a, C2, C2a and C1 (Fig. S3), was obtained from Zhichu Pharmaceutical Factory (ZPF) in Shandong Province, China. The chromatographic grade reagents, including methanol purchased from Fisher Scientific (Massachusetts, USA) and trifluoroacetic acid (TFA) purchased from Bailingwei Science and Technology (Beijing, China), were used for the detection of gentamicin. Ultrapure water was prepared by a Milli-Q Advantage A10 system from Millipore (Massachusetts, USA). All the other chemicals obtained from Wanxin Company (Beijing, China) were of high purity and analytical grade.

### Sampling

Four kinds of wastes arising from gentamicin production, including QD1, QD2, QD3 and QD4, were collected and conveyed in ice-boxes from ZPF to laboratory in Beijing. Then they were preserved in refrigerator (4 °C) for the domestication and isolating of gentamicin-degrading bacteria consortia. In addition, raw gentamicin sewage, which was obtained by centrifuging the gentamicin waste residue and collecting the suspension, was stored in refrigerator (4 °C) to confirm gentamicin degradation efficiency by the isolated microflora.

### Development of gentamicin-degrading bacterial consortia

To domesticate gentamicin-degrading bacterial consortia, 5.0 g of four different wastes in triplicate were dissolved in 100 ml of sterile water in 250-ml Erlenmeyer flasks respectively, followed by adding appropriate aseptic glass beads. The flasks were vibrated in a rotary shaker at 30 °C for 30 minutes to scatter the samples. Then 1.0 ml of the four lixivium samples (named as initial samples) with three replicates were separately added to flasks with 99.0 ml of BEP (beef extract, 3.0 g, peptone, 5.0 g, NaCl 1.0 g, and ultrapure water 1,000 ml) supplemented with 50.0 mg L^−1^ of gentamicin and were incubated in an incubator (30 °C, 150 r/min). One week later, 1.0 ml of fermentation liquor from each replicate was transferred to a 250-ml Erlenmeyer flask containing 99.0 ml of half concentration of BEP and 100.0 mg L^−1^ of gentamicin and was sub-cultured at 30 °C and 150 r/min. Similarly, 1.0 ml of suspension from each medium cultured one week was added to flasks with 99.0 ml of 1/5 of BEP containing 150.0 mg L^−1^ of gentamicin for another hebdomadal acclimatization. Then 1.0 ml of each medium was mixed with 99.0 ml of fresh 1/10 of BEP with 200.0 mg L^−1^ of gentamicin. These flasks were continually cultivated in at 30 °C and 150 r/min for one week to get the final domesticated microflora. The whole process of domestication was lasted four weeks.

To evaluate the gentamicin degradation ability of initial samples and the acclimatized mixed microbe, four kinds of initial lixivium and domesticated suspension were inoculated in 100.0 ml of 1/5 of BEP with 100.0 mg L^−1^ of gentamicin in the inoculum of 1% (v/v). To investigate whether the microflora could use gentamicin as the sole carbon source, they were also added to 100.0 ml of mineral salt medium (MSM) with 100.0 mg L^−1^ of gentamicin in the inoculum of 1% (v/v). The standardized inocula were prepared as described by Jabeen *et al*. and 1% of inoculum was equivalent to 8.2 × 10^8^ colony forming units (CFU). In this study, all the Erlenmeyer flasks were under wraps of tinfoil to avoid photodegradation of gentamicin. And uninoculated medium was used as the control treatment. Each treatment was performed in triplicate.

### Optimization of gentamicin degradation

The initiatory studies showed that AMQD4 has the strongest ability to degrade gentamicin. So AMQD4 was used for the optimization of gentamicin degradation with single factor tests. The parameters including medium concentration (1/1–1/20), initial gentamicin concentration (50–400 mg L^−1^), shaking frequency (90–210 r/min), inoculum size (1–10%), initial pH (4.0–10.0), temperature (20–60 °C), salinity (0.0–5.0%) and liquid volume (60–160 ml in 250-ml flasks) were optimized. The eight factors were tested one after another. The condition that enabled the highest gentamicin removal was used for the optimization of the next parameter. For example, in order to evaluate the effect of the BEP medium concentration on gentamicin degradation by AMQD4, 1% of AMQD4 was added into 100 ml of 1/1, 1/5, 1/10, 1/15, 1/20 diluted BEP medium at pH 6 with 100 mg L^−1^ gentamicin, and was incubated for 7 days at 30 °C and 150 r/min. Then gentamicin concentration was optimized at the BEP medium concentration that gave the highest gentamicin removal, while other parameters were maintained unchanged. Other parameters were similarly optimized.

### Analytical determinations

#### Biomass estimation

The growth of AMQD4 was determined by detecting OD600 with spectrophotometer^32^. Before the detection of OD600, the apparatus was set to zero with the control without bacteria.

#### Extraction and analysis of gentamicin residues

Shake up the culture medium containing gentamicin and transfer 20 ml of the medium into a 50 ml of centrifuge tube. Then adjust the pH of the culture medium to 4.5 with 0.1 M HCl. 1.0 g resin was added into the medium. To achieve a better absorption of gentamicin to resin, the centrifuge tube was vibrated in a shaker at 30 °C for 2 h. The supernatant liquid was discarded and the resin was washed thrice with 5 ml of water: methanol (90:10) solution, following by desorption with 20 ml of 4.5% ammonia water for 4 h in a shaker at 30 °C. The suspension was collected into a round-bottom flask and was evaporated to dryness at 45 °C with rotary evaporator. The residue was dissolved completely with 2 ml of ultrapure water and was filtered through 0.22 μm sterile membrane to determine the gentamicin concentration as the method described in China Medicine and Pharmacy^33^.

The quantification of gentamicin was expressed as a concentration percentage which was computed based on the peak area of standard solutions. The degradation efficiency (*D*) was calculated using the following formulas:

$$D=[({C}_{{1}}-{C}_{{2}})-({C}_{{1}}-{C}_{{3}})]{{C}_{{1}}}^{-1}\times 100 \% =({C}_{{2}}-{C}_{{3}}){{C}_{{1}}}^{-1}\times 100 \% ;$$

where: *C*_*1*_: The initial concentration of gentamicin in the fermentation broth; *C*_*2*_: The concentration of gentamicin in the control at the end of fermentation; *C*_*3*_: The concentration of gentamicin in the spent medium at the end of fermentation.

### Application of AMQD4 in the degradation of gentamicin in raw gentamicin sewage

The physicochemical characteristics of raw gentamicin sewage obtained from ZPF are presented in Table 2. The experiment was conducted on 4 treatments: (1) sterilized sewage without AMQD4 (SSWA); (2) sterilized sewage +2.5% AMQD4 (SSA); (3) unsterilized sewage without AMQD4 (USWA); (4) unsterilized sewage +2.5% AMQD4 (USA). The sterilized sewage was obtained by autoclaved sterilization at 121 °C for 20 min in the autoclave. And our trail test has proved that gentamicin did not degrade in the autoclave. Similar to the optimized conditions, the 250-ml Erlenmeyer flasks loaded with 100 ml sewage were incubated at 40 °C and 110 r/min orbital shaking for 7 days. Then concentration of gentamicin and OD600 of the fermentation broth were also detected. And each treatment was replicated three times.

**Table 2:** Physicochemical characteristics of the raw gentamicin sewage.

Parameters	Value
Total Carbohydrate(g L^−1^)	2.80 ± 0.04
Amino nitrogen(g L^−1^)	0.84 ± 0.05
pH	6.40 ± 0.4
COD (g L^−1^)	14.38 ± 0.51
Gentamicin (mg L^−1^)	35.91 ± 6.63

### Identification of the isolated bacteria

The isolates obtained from AMQD4 were grown on BEP medium for 36 h at 30 °C. Then 16S rDNA of the isolates was amplified as previously described (Islas-Espinoza *et al*.^9^) and agarosegel electrophoresis was run to check amplicon purity and appropriate size. The purified products were sent to Majorbio Bio-Pharm Technology Company (Beijing, China) for sequencing. The original sequences were submitted to Genbank and compared with the NCBI database using BLASTA.

### Gentamicin degradation efficiencies of the isolated bacteria

Gentamicin degradation efficiencies of the four isolates obtained from AMQD4 were evaluated in the optimized condition.

### Identification of degradation genes

The best gentamicin degrader Pseudomonas *diminuta* BZC3 isolated from AMQD4 and *Pseudomonas diminuta* ACCC10507, without gentamicin degradation ability, obtained from Agricultural Culture Collection of China (ACCC) were sent to Majorbio Bio-Pharm Technology Company (Beijing, China) for genome sequences using Illumina HiSeq sequencing platform. After the raw data is filtered to clean data, SOAPdenovo software^34^ was used to construct a fine and draft genome for *Pseudomonas diminuta* BZC3 and *Pseudomonas diminuta* ACCC10507, respectively. Then gene prediction was performed by GeneMarkS in version 4.6b and genomic functions were annotated by ARDB (http://ardb.cbcb.umd.edu/)^35^. The different antibiotic resistance genes of the two bacteria were compared for the identification of degradation genes. Genome sequence data have been deposited at DDBJ/ENA/GenBank under the accessions CP021995 and NIVP00000000, respectively. The version of *Pseudomonas diminuta* ACCC10507 described in this paper is version NIVP01000000.

### Nucleotide sequence accession numbers

Sequences of the isolated bacteria have been submitted to GenBank with accession number of KU984707 (BZC1), KU306965 (BZC3), KU984708 (BZC5), and KU984709 (BZC6).

### Statistical analysis

Statistical analyses were performed with SPSS Statistics ver. 19 software. The significant difference between different treatments were determined using one-way analysis of variance (ANOVA) and the differences between individual means were calculated using Duncan’s multiple range test.

## Electronic supplementary material

Supplementary materials
