# Supplementary material for: Biodegradation of gentamicin by bacterial consortia AMQD4 in synthetic medium and raw gentamicin sewage
Source: Sci Rep. 2017 Sep 8;7:11004. doi: 10.1038/s41598-017-11529-x (PMC5591267; doi:10.1038/s41598-017-11529-x)
Supplement: Supplementary file 1 — Supplementary materials [file 41598_2017_11529_MOESM1_ESM.pdf]

Supplementary material

Biodegradation of gentamicin by bacterial consortium AMQD4 in synthetic medium and raw gentamicin sewage

Yuanwang Liu[1], Huiqing Chang[2], Zhaojun Li[1,*], Yao Feng[1], Dengmiao Cheng[1], Jianming Xue[3]

[1]Institute of Agricultural Resources and Regional Planning, Chinese Academy of Agricultural Sciences, Key Laboratory of Plant Nutrition and Fertilizer, Ministry of Agriculture, Beijing, 100081, China

[2]Henan University of Science and Technology, Luoyang, 471003, China

[3]Scion, Christchurch 29-237, New Zealand

[*]Corresponding author. Tel: +86 82108657. Fax: +86 10 8210 9640. E-mail: lizhaojun@caas.cn.

This supporting information contains two figures and their captions. The detailed descriptions of them are showed in the manuscript.

Table S1 General genome properties of Brevundimonas diminuta BZC3 and Brevundimonas diminuta ACCC10507.

Fig. S1 The effect of different culture conditions on final pH value at the end of fermentation (**a**, medium concentration; **b**, gentamicin concentration; **c**, shaking frequency; **d**, inoculum size; **e**, initial pH value; **f**, temperature; **g**, salinity; **h**, liquid volume). The mean values and SD (*error bars*) from triplicate trials are presented. *Data bars* having the same letter are not significantly different from each other at the 95 % confidence level in the Duncan's test ($P< 0.05$).

Fig. S2 The effect of different culture conditions on AMQD4 growth (**a**, medium concentration; **b**, gentamicin concentration; **c**, shaking frequency; **d**, inoculum size; **e**, initial pH value; **f**, temperature; **g**, salinity; **h**, liquid volume). The mean values and SD (*error bars*) from triplicate trials are presented. *Data bars* having the same letter are not significantly different from each other at the 95 % confidence level in the Duncan's test ($P< 0.05$).

Fig. S3 Gentamicin structure graphing. Gentamicin C component: C1 (R1, CH3; R2, H; R3, CH3), C1a (R1, H; R2, H; R3, H), C2 (R1, H; R2, H; R3, CH3) and C2a (R1, H; R2, CH3; R3, H).

Table S1 General genome properties of *Brevundimonas diminuta* BZC3 and Brevundimonas diminuta ACCC10507

Genomic Characteristic	BZC3	ACCC10507
Genome size (bp)	3,385,620	3,402,991
Gene average length (bp)	928	903
Predicted CDS (bp)	3279	3342
CDS dinsity	89.87	88.64
Number of contigs	6	57
N50 (bp)	1,027,743	128,559
Largest Contig	1,310,254	309,624
GC content (%)	67.37	67.31
Number of tRNAs	52	50
Number of rRNAs	6	3

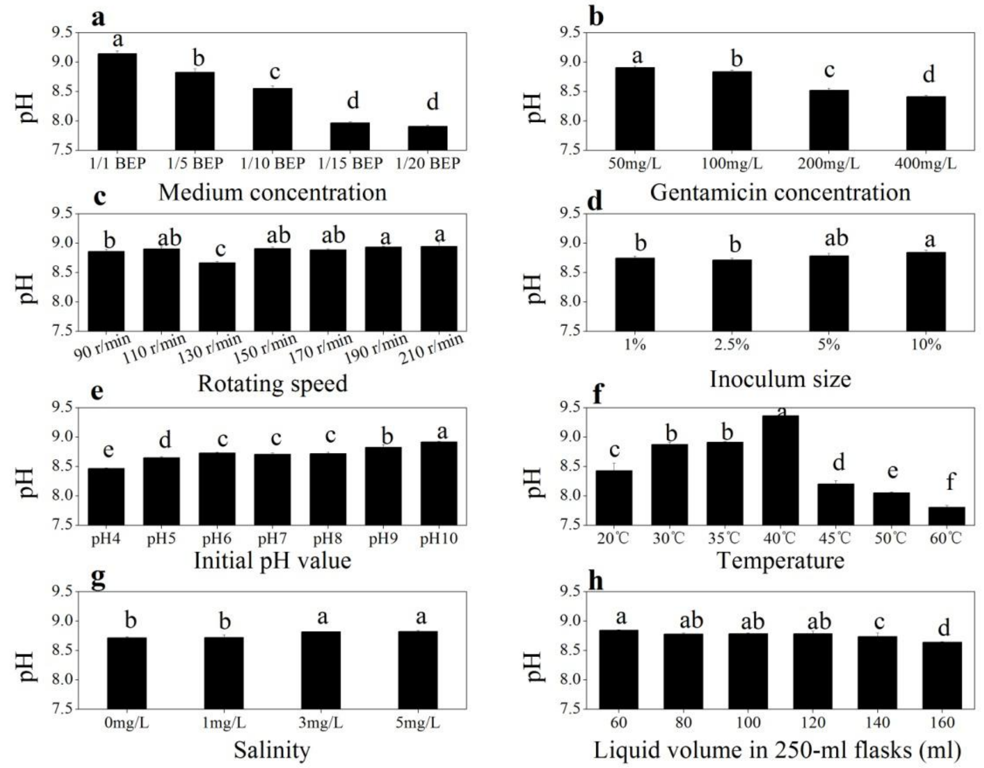
a
b
c
d
e
f
g
h
pH
9.5
9.0
8.5
8.0
7.5
1/1 BEP
1/5 BEP
1/10 BEP
1/15 BEP
1/20 BEP
Medium concentration
50mg/L
100mg/L
200mg/L
400mg/L
Gentamicin concentration
90 r/min
110 r/min
130 r/min
150 r/min
170 r/min
190 r/min
210 r/min
Rotating speed
1%
2.5%
5%
10%
Inoculum size
pH4
pH5
pH6
pH7
pH8
pH9
pH10
Initial pH value
20℃
30℃
35℃
40℃
45℃
50℃
60℃
Temperature
0mg/L
1mg/L
3mg/L
5mg/L
Salinity
60
80
100
120
140
160
Liquid volume in 250-ml flasks (ml)

Fig. S1

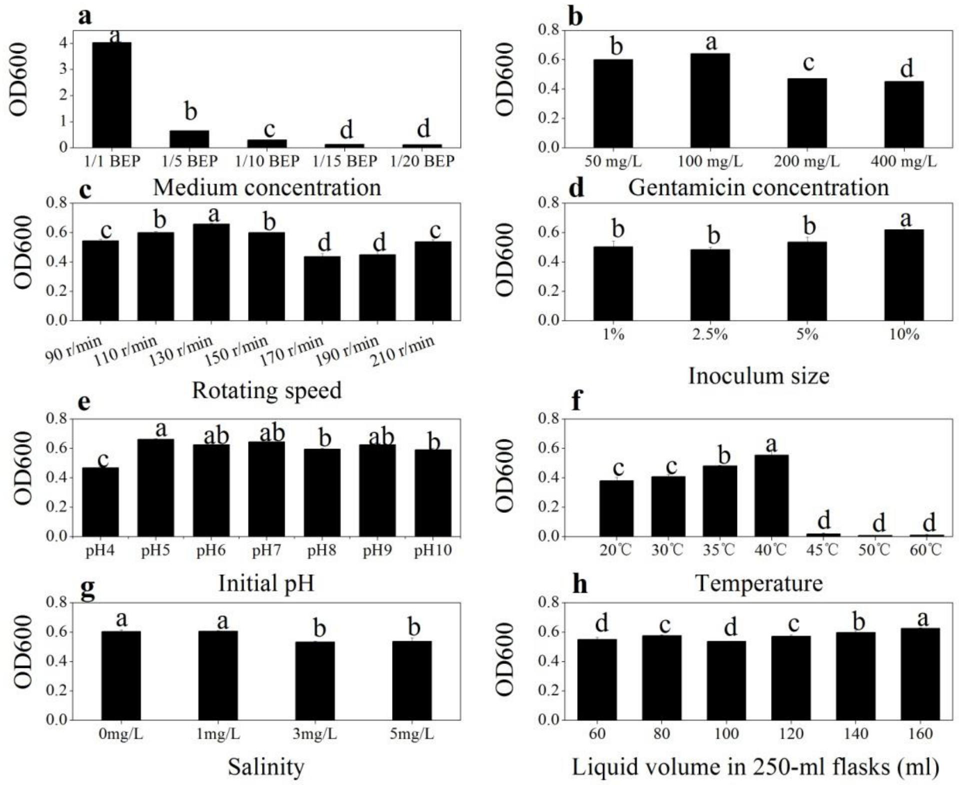
a
OD600
1/1 BEP
1/5 BEP
1/10 BEP
1/15 BEP
1/20 BEP
Medium concentration
b
50 mg/L
100 mg/L
200 mg/L
400 mg/L
Gentamicin concentration
c
90 r/min
110 r/min
130 r/min
150 r/min
170 r/min
190 r/min
210 r/min
Rotating speed
d
1%
2.5%
5%
10%
Inoculum size
e
pH4
pH5
pH6
pH7
pH8
pH9
pH10
Initial pH
f
20℃
30℃
35℃
40℃
45℃
50℃
60℃
Temperature
g
0mg/L
1mg/L
3mg/L
5mg/L
Salinity
h
60
80
100
120
140
160
Liquid volume in 250-ml flasks (ml)

Fig. S2

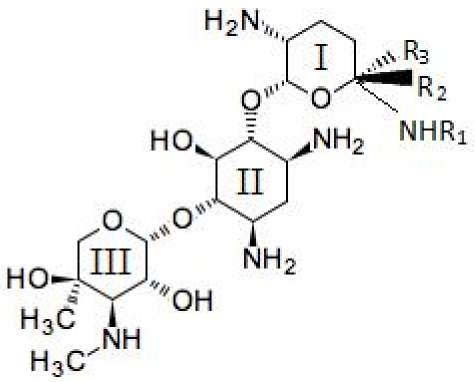

Fig. S3
